# A Reliable System for Quantitative G-Protein Activation Imaging in Cancer Cells

**DOI:** 10.3390/cells13131114

**Published:** 2024-06-27

**Authors:** Elena Mandrou, Peter A. Thomason, Peggy I. Paschke, Nikki R. Paul, Luke Tweedy, Robert H. Insall

**Affiliations:** 1 CRUK Scotland Institute, Garscube Campus, Glasgow G61 1BD, UK; 2 School of Cancer Sciences, University of Glasgow, Glasgow G61 1QH, UK; 3 Division of Cell & Developmental Biology, University College London, London WC1E 6BT, UK

**Keywords:** G-protein signalling, FLIM, FRET, GPCR, LPA, LPAR, live cell imaging, PDAC, fluorescence lifetime

## Abstract

Fluorescence resonance energy transfer (FRET) biosensors have proven to be an indispensable tool in cell biology and, more specifically, in the study of G-protein signalling. The best method of measuring the activation status or FRET state of a biosensor is often fluorescence lifetime imaging microscopy (FLIM), as it does away with many disadvantages inherent to fluorescence intensity-based methods and is easily quantitated. Despite the significant potential, there is a lack of reliable FLIM-FRET biosensors, and the data processing and analysis workflows reported previously face reproducibility challenges. Here, we established a system in live primary mouse pancreatic ductal adenocarcinoma cells, where we can detect the activation of an mNeonGreen-Gαi3-mCherry-Gγ2 biosensor through the lysophosphatidic acid receptor (LPAR) with 2-photon time-correlated single-photon counting (TCSPC) FLIM. This combination gave a superior signal to the commonly used mTurquoise2-mVenus G-protein biosensor. This system has potential as a platform for drug screening, or to answer basic cell biology questions in the field of G-protein signalling.

## 1. Introduction

Since fluorescence resonance energy transfer (FRET) was first described [1] and its potential was appreciated in cell biology, a wide variety of biosensors have been designed for detecting or measuring biological events of interest. Energy transfer between a donor and acceptor fluorophore linked to proteins is most commonly measured either through changes in their fluorescence intensity (intensity-based FRET) or, alternatively, using fluorescence lifetime imaging microscopy (FLIM).

Fluorescence lifetime (tau; τ) is defined by the mean amount of time a fluorophore remains in the excited state after being stimulated by an appropriate wavelength and is usually measured in nanoseconds or picoseconds. The advantages of using FLIM to measure FRET over fluorescence intensity-based methods, as well as the benefits of two-photon time-correlated single-photon counting (TCSPC) FLIM used in this study, have been extensively reviewed in the literature [2,3,4,5,6]. It is, therefore, not surprising that the FLIM biosensor toolkit has expanded [7,8,9], which together with the engineering of brighter FLIM-appropriate donor fluorescent proteins like mNeonGreen [10,11] and advances in electronics (lasers, scanners and detectors), have added to the possibilities, and the complexity, of experiments.

However, because of its intrinsic technical difficulty, research involving FLIM biosensors can be hard to reproduce and can lack transparency when it comes to data processing, analysis and visualisation. Reproducibility and transparency are particularly important considerations given the challenges inherent to FLIM-FRET experiments on G-protein signalling, and there are several ways in which methods can vary between research groups:

1. The equipment. FLIM systems may be from one of several sources, or even be custom-built from individual parts;
2. The software. Analysis tools may be free [12], may be proprietary or may have been developed in-house and used only by one group;
3. The cells. Cell signalling itself can be remarkably heterogeneous within a cell population [13,14], or across different cell lines, and experiments frequently require the overexpression of a G-protein-coupled receptor (GPCR) that is not normally expressed at experimentally tractable levels, or even at all [15];
4. The effect size. G-protein activation does not necessarily lead to a complete subunit dissociation and, thus, a massive loss of FRET, but can rather be a more subtle rearrangement [16,17,18], which requires higher sensitivity in the detection of FRET efficiency changes.

There is a substantial gap in the current literature surrounding cell models that can be used for clinically relevant in vitro experiments, rather than commonly used cell lines such as HEK293 cells or MDCK. We, therefore, used more directly cancer-relevant pancreatic cancer cells, despite the resultant need for extensive transfection optimisation. In particular, previous work implies that chemotaxis towards lysophosphatidic acid (LPA) is a principal feature of metastasis in patients with melanoma [19] and pancreatic ductal adenocarcinoma (PDAC; [20]). In these patients, metastasis is the principal cause of morbidity and death [21]. To our knowledge, there is no current system for quantitative measurement of G-protein activation in primary cancer cells in response to LPA stimulation through the G-protein-coupled receptors LPAR1/3 and the associated Gαi subunit [22]. The components of this signalling axis are thought to be key transducers of GPCR-mediated chemotaxis and are some of the most potent regulators of localised actin polymerisation [23]. There is plainly an unmet need for reporters of LPA signalling in live cells or tissues.

In this work, we probed LPA signalling in primary mouse pancreatic ductal adenocarcinoma (PDAC) cells through its cognate LPAR1/3 [20,23,24] by adapting an existing Gαi3-Gβ1γ2 biosensor described by van Unen et al. (2016) [15] by replacing the mTurquoise2-mVenus donor–acceptor fluorescent protein pair with mNeonGreen-mCherry, otherwise retaining their original position in the sequence.

We show that in live primary mouse PDAC cells [25], the mNeonGreen-mCherry G-protein biosensor has well-defined membrane localisation and is a brighter alternative to the mTurquoise2-mVenus option, making the detection of small FRET changes detectable with 2-photon TCSPC FLIM. The FLIM data presented here were extracted using FLIMfit (open-source software) [12] and were visualised with SuperPlotsofData, a free web app that encourages transparency by displaying all data points of each replicate [26]. Finally, since FLIMfit is a complex tool, we go into detail about the parameters used to fit the data in order to emphatically promote best practices in future studies dealing with FLIM data.

## 2. Materials and Methods

### 2.1. PDAC Cell Culture and Transient Transfection

PDAC cells were a kind gift from Professor Jennifer Morton (Kras^G12D^–p^53R175H^-Cre genotype) [25]. They were cultured in DMEM supplemented with 10% FCS, 1% penicillin/streptomycin and 1% L-glutamine, at 37 °C, 5% CO_2_. The cells were tested for mycoplasma to ensure they were negative. The cells were transiently transfected using 3 μg DNA with the Nucleofector™ (Lonza Group AG, Basel, Switzerland) Kit V (program P-031) according to the manufacturer’s instructions the morning of the day before imaging. The cells were plated in 35 mm glass-bottom dishes (MatTek, Ashland, MA, USA) in the abovementioned DMEM formulation omitting penicillin/streptomycin. After attachment (4–6 h later), cells were serum-starved overnight in medium containing Fluorobrite (Thermo Fisher Scientific, Waltham, MA, USA) supplemented with 1% L-glutamine and 1% sodium pyruvate (starvation media).

The next morning, before imaging, transfected cell samples were washed with PBS, and the medium was replaced with fresh starvation media. An aliquot was taken for the control stimulation that would be used during imaging. A solution of starvation medium supplemented with 10% fresh FCS for sample stimulation was also prepared. For the LPA stimulation solution, starvation medium was mixed with LPA at 3 μΜ final concentration, or DMSO for control equivolume to the Ki16425 used for the LPAR1/3 inhibition experiments.

In all LPA stimulation solutions, 0.5% fatty-acid-free BSA in PBS was incorporated as a carrier. 

### 2.2. Plasmid Construction

To create the mNeonGreen-mCherry G-protein biosensor, a four-fragment Gibson Assembly (NEB; according to the manufacturer’s instructions) was carried out, and the mTurquoise2-mVenus biosensor (acquired from Addgene 69625; [15]) was used as a starting point and was digested with SgrAI and SacI. All four fragments were obtained by standard PCR. 

The mCherry and mNeonGreen fragments were acquired through PCR from two plasmids, which were a gift from the group of Prof. Laura Machesky (CRUK Scotland Institute/University of Cambridge), by using the primer pairs 101 and 102, and 103 and 104, respectively. Note that any mNeonGreen and mCherry plasmids would work for this Gibson Assembly. 

The third fragment, which codes for the C-terminal part of the Gβ subunit + T2A, and the fourth fragment coding for the Gγ + IRES + N-terminal of the Gα subunit were obtained from the 69,625 plasmid (Addgene; https://www.addgene.org/) by PCR using the primer pairs 105 and 106, and 107 and 108, respectively. 

### 2.3. Cell Handling before and during Imaging

After pre-stimulation images of live cells were taken, 500 μL (out of 1.5 mL) of medium was carefully taken out of the dish and discarded without disturbing the sample on the stage, and 500 μL of starvation medium with LPA (final concentration in the sample was 1 μΜ), 10% FCS (final concentration in the sample was 3.3%), DMSO or plain starvation medium was added and carefully resuspended ~3 times. For experiments using the Ki16425 LPAR1/3 inhibitor, cells were incubated 45–60 min with either 10 μM or 100 μM of the inhibitor (final concentration) prior to imaging. Control samples were incubated with DMSO (Ki16425 vehicle) instead.

### 2.4. Instrumentation

All FLIM image acquisition was carried out on a LaVision Biotec (Bielefeld, Germany) TriMScope system equipped with a FLIM χ16 bioimaging detector (LaVision Biotec; FLX-D-0003). For excitation, a Chameleon Ultra II (Coherent, Saxonburg, PA, USA) femtosecond laser was used. The microscope body was a Nikon Eclipse TE2000-U, with a Nikon Apo 60× 1.40 NA oil immersion lens. A 525/50 BrightLine (Semrock; IDEX corp., Northbrook, IL, USA) and a ET480/40 (Chroma; Chroma Technology Corp., Bellows Falls, VT, USA) filter were used for mNeonGreen or mTurquoise2 detection, respectively. The image acquisition software for the LaVision Biotec TRIMscope system was Imspector pro (v. 7). The microscope was fitted with a temperature- and CO_2_-controlled chamber.

An Instrument Response Function (IRF) measurement was always taken using a sample of immobilised gold nanorods, using the 970 nm or 890 nm wavelength (depending on fluorophore imaged), at 1% laser power and a 10-line average. The gold nanorod sample was a gift by Paul French (Imperial College London), whose preparation was previously described by Talbot et al. (2011) [27].

### 2.5. Quantitative Lifetime Data Extraction Using FLIMfit

Raw FLIM-FRET data were imported into FLIMfit (version 5.1.1) as .msr files. Smoothing was set to 5 × 5 (B&H 2), TVB under the Stray Light tab was set to 1, and the IRF image was imported using the top IRF tab > Load IRF. Because the intensity decay decreases exponentially approximating 0, there will be an artificial cut-off at the end of the tail. For a clean cut-off, the Time Max value under the Data and IRF tab was set to 1.155 × 10^4^. The square region of interest selection tool was used to select a cell-free area with no signal (i.e., a flat intensity decay), and under the top Background tab “Use Selected Region as Time-Varying Background” was selected. This was done to account for sample-specific background signal and autofluorescence. Then, under the Data tab, the Integrated Min value was set to 35. To segment the cell membranes, under the Segmentation tab, the Segmentation Manager was selected. With the brush tool at brush width 4, all cell membranes of alive, healthy and in-focus cells were drawn and saved. Leaving all other fitting parameters at default, the dataset is fitted (with a single exponential). The exported “Fit Results Table” .csv file contains the quantitative measurements of fluorescence lifetimes.

### 2.6. Data Filtering

The quality of data present in the “Fit Results Table” .csv files was assessed based on two parameters—the mean chi-squared value (goodness of fit) and the mean intensity value. Data filtering was conducted in Excel (v. 16.86). Acceptable mean chi-squared values were considered as all those that were under 1.4. This indicates how well the sample intensity decay matches with the model fit, with an ideal value being 1. It is highly important to note that with complex biological cell samples (unlike pure fluorescein in solution for instance), an ideal chi squared of 1 is somewhat infrequent. There could be any number of reasons behind this, which are currently not fully understood and rarely discussed in the literature. Nevertheless, if one’s samples are characterised by too many chi-squared values above 1.4, we emphasise that this is not a reason to fit the data with two exponentials. Uniquely, the reasons behind that have been extensively discussed by Lee et al. (2001) [28].

It is frequently stated in the literature that FLIM is independent of fluorophore concentration and abundance [3]. However, this may only be true in physics theory and only applies to simple experimental systems like dyes in solutions. When complex biological samples are imaged, intensity plays a role. If the sample is too dim, the lifetime measurement becomes inaccurate and, thus, cannot be trusted. Empirically, the researcher must find an intensity threshold for which lifetime measurements are trustworthy. In our case, at the mean intensity column of the Fit Results Table, if there is a significant number of values under 50, the bottom 15% dimmest cells are filtered out of the pre-stimulation images. Then, the highest-intensity value of those 15% dimmest is used to filter out the dimmest post-stimulation cells. Of course, each pre- and post-stimulation cell is paired; thus, if there is a case where a post-stimulation cell is too dim but its pre-stimulation state was not filtered out, both will be deleted to preserve the symmetry of the data.

### 2.7. Data Visualisation and Statistical Analysis

Filtered data were imported into a Python-integrated development environment.

The packages pandas (v. 2.1.4), matplotlib (v. 3.8.0), statannot (v. 0.2.3) and seaborn (v. 0.12.2) were used to create the graphs.

The statannot package was also used to obtain statistical information for the independent *t*-tests.

The equation used to obtain the Cohen’s d values, as well as how to interpret them, was based on the publications by Brownlee (2019), Cohen (1988), Lakens (2013) [29,30,31].

To format the data for upload into SuperPlotsofData [26], code was written to subtract the post-stimulation from the pre-stimulation lifetimes so that a lifetime difference plot would be generated.

## 3. Results

### 3.1. The mNeonGreen-mCherry G-Protein Biosensor Is a Brighter Alternative to mTurquoise2-mVenus

**Figure 1:**
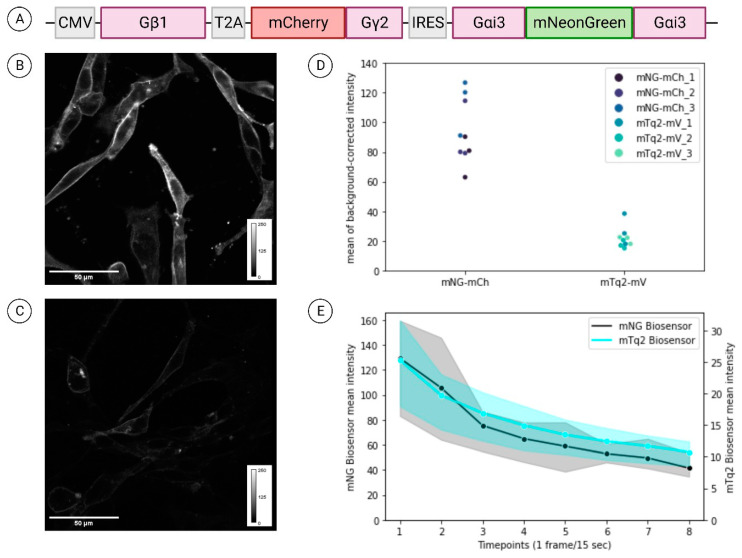
Comparison of signal intensity and photobleaching between the mNeonGreen- mCherry and the mTurquoise2-mVenus G-Protein Biosensors. (**A**) Schematic of the mNeonGreen- mCherry biosensor plasmid expressing all three subunits of the heterotrimeric G-protein complex under the control of the CMV promoter to achieve similar expression levels. Representative signal intensity images of PDAC cells transiently transfected with the (**B**) mNeonGreen-mCherry and (**C**) mTurquoise2-mVenus biosensor taken with our 2-photon TCSPC FLIM system (TriMScope). In both (**B**,**C**), intensity scales are shown on the bottom right. (**D**) Quantification of signal intensity comparisons between the two biosensors. Signal intensity was measured on three different days for each biosensor (n = 3), with 3 cells per biosensor quantified each day (n = 9 datapoints per biosensor). (**E**) Quantification of photobleaching for the two biosensors. Solid lines represent the mean signal, while the shaded area represents the range of values of three replicates. In both (**D**,**E**), y-axes show arbitrary intensity units.

In the current FRET literature, mTurquoise2 is frequently presented as the brightest (with the cyan fluorescent protein range specification sometimes omitted) and most effective option as a donor fluorophore [32,33,34]. During the optimisation of the biosensor, our observations consistently disagreed with this assessment. Thus, we tested whether the original mTurquoise2-mVenus Gαi3-Gβ1γ2 biosensor, made by van Unen et al. (2016) [15], was optimal, by replacing the fluorophore pair with the *Branchiostoma lanceolatum*-derived mNeonGreen as a donor, and the *Discosoma* sp.-derived mCherry as the acceptor [11,35]—a pair whose benefits for FLIM-FRET applications were first reported by McCullock et al. (2020) [10]. Our data show the substantial signal intensity difference between the mNeonGreen-mCherry (Figure 1A) and mTurquoise2-mVenus G-protein biosensors, where the former is approximately four-times brighter than the latter (Figure 1B–D). Usual techniques of improving signal output during acquisition, such as increasing laser power or number of line average, are typically not viable for mTurquoise2 due to the high energy of the 890 nm laser wavelength. Therefore, the laser power must be carefully chosen so that maximal signal is extracted (for accurate lifetime measurements) without damaging the cells. In this study, for the PDAC cells shown in Figure 1B,C, we used 20% and 12% laser power to excite mNeonGreen and mTurquoise2, respectively. Cells tolerated the 20% power for mNeonGreen well since the 970 nm laser has lower energy than the 890 nm wavelength laser, making it less phototoxic. Despite the fact that only two images were acquired per field of view [36], we were also interested in comparing the photostability of the two different donor fluorescent proteins. The data in Figure 1E show that mTurquoise2 photobleached more slowly than mNeonGreen. However, this was not practically relevant, as we found it never reached the threshold of signal intensity needed to measure fluorescence lifetimes with acceptable accuracy. 

Finally, two further factors should be noted that were taken into account when selecting fluorescent protein pairs—the donor emission and acceptor excitation spectral overlap, and the maturation times. Both fluorophore pairs have been previously shown to have similar overlap integrals (J(λ)), with that of mTurquoise2-mVenus, being reported to be 2.3 (×10^−15^ M^−1^cm^−1^nm^4^) and 2.28 (×10^−15^ M^−1^cm^−1^nm^4^) for mNeonGreen-mCherry [10,37]. Regarding the maturation time, it is desirable for each donor–acceptor pair to fold and mature within a comparable timeframe, while faster maturation times obviously favour the efficiency of energy transfer [2,38]. mNeonGreen and mCherry display favourable maturation times at 10 and 15 min, respectively, while mTurquoise2 takes longer to fold and almost double the time of mVenus (33.5 and 17.6 min, respectively) [10,39]. This may contribute to the low brightness of mTurquoise2 that we have experienced.

### 3.2. FLIM-FRET Shows Activation of the mNeonGreen-mCherry G-Protein Biosensor after Lysophosphatidic Acid or Serum Stimulation in Pancreatic Cancer Cells

Initially, to test the mNeonGreen-mCherry G-protein biosensor, we stimulated transiently transfected PDAC cells with a final concentration of 3.3% fetal calf serum (FCS) in phenol red-free medium (Fluorobrite). Most cells reacted immediately and obviously, losing attachments to the glass-bottom dish, making multiple blebs and retracting their edges, as expected, after stimulation by the LPA present in the serum [40]. The biosensor’s fluorescence lifetimes (τ) were measured before and after stimulation using 2-photon TCSPC (TrimScope), counting only segmented plasma membranes where active receptors are located. It should be noted here that when stimulating cells with pure LPA, a carrier is needed to ensure the lipid’s solubility and delivery to the cells. We and others [41] have used bovine serum albumin (BSA) as a carrier for LPA, since that is one of its natural functions [42].

**Figure 2:**
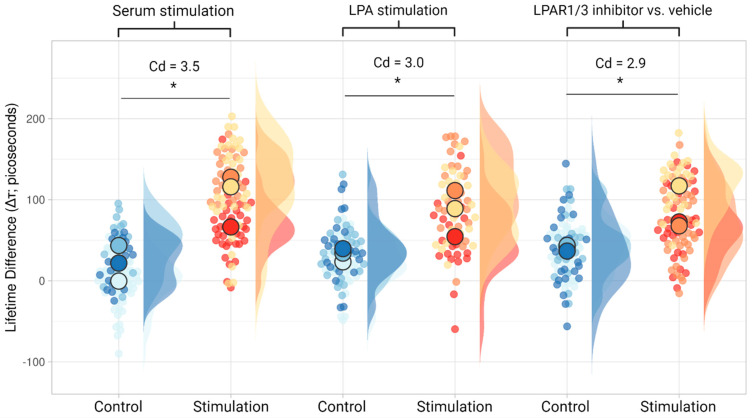
The mNeonGreen-mCherry G-protein biosensor is activated by serum (FCS; fetal calf serum), and LPA. For serum stimulation, a final concentration of medium with 3.3% FCS was used. The final concentration of the LPAR1/3 inhibitor (Ki16425) used was 10 μM. Final LPA concentration used was 1 μM. The independent *t*-test was used to calculate statistical significance with the statannot Python package (*: 1.00 × 10^−2^ < *p* ≤ 5.00 × 10^−2^). The *p* values for the serum stimulation, LPA stimulation, and inhibitor vs. vehicle are 0.02, 0.03, and 0.04, respectively. The data visualisation web tool SuperPlotsofData [26] was used to present the data. The different colours used show separate replicates, with the larger dot indicating the mean. Distribution per replicate is also shown. Cohen’s d (Cd) was calculated with the numpy (v. 1.26.4) package in Python, and reports the effect size. LPA; lysophosphatidic acid. The inhibitor vehicle was dimethylsulfoxide (DMSO).

However, we found during the optimisation of the assay that commercial BSA was already bound to lipid signalling molecules such as LPA, giving an artefactual change in unstimulated control cells. We, therefore, used fatty acid-free (FAF) BSA as a carrier. Figure 2 shows the difference between lifetimes before and after the addition of either a stimulatory or a control solution. Control stimuli containing no active lipids (Fluorobrite only, or Fluorobrite plus 0.5% FAF BSA) did not lead to biologically relevant lifetime changes. On the other hand, stimulatory solutions containing FCS or LPA led to a significant lifetime increase, indicating Gαi activation. Finally, we confirmed that LPAR1/3 are the main LPA signal transducers by showing a lack of G-protein biosensor activation when the specific LPAR1/3 inhibitor, Ki16425, and LPA were combined.

We also recommend the reporting of the Cohen’s d value alongside the independent *t*-test used when analysing the Δτ data (Figure 2). Note that the use of Δτ as a means of comparison between conditions is more meaningful and biologically relevant than the absolute pre- and post-intervention lifetimes. Accordingly, Cohen’s d is a measure of effect size, denoting “the degree to which the null hypothesis is false”, where 0.2, 0.5 and >0.8 signify a small, medium and large effect, respectively [30,43]. It is calculated by dividing the difference between the means of the two populations by the pooled standard deviation and can also be interpreted as a difference in terms of a percentage of the standard deviation [30,31]. For example, within the context of Figure 2, this means that the difference between the “Control” and “Stimulation” in the “Serum Stimulation” samples is equal to three and a half standard deviations, which is a bigger lifetime difference than what was observed in the “LPA stimulation” and “LPAR1/3 inhibitor vs. vehicle” samples. This implies that, perhaps unsurprisingly, there are moieties in serum (apart from LPA) that can activate additional Gαi3-coupled receptors. Cohen’s d does not measure the amount or physiological significance of the difference—that is a nuanced decision requiring biologists, rather than statistics—but it gives clarity about whether the effect is robust. Thus, we advocate for the use of this measure in order to enhance the meaning of the *p*-value, since the latter only reports whether there is any difference between two populations and does not answer the question of “to what degree?” [44].

**Figure 3:**
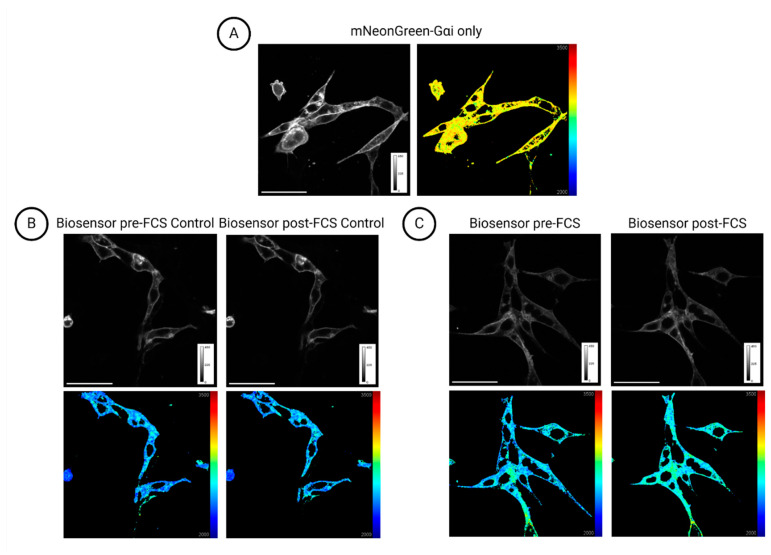
Examples of signal intensity (in grayscale) and false-coloured lifetime images. (**A**) PDAC cells expressing mNeonGreen-Gαi3 only. (**B**,**C**) PDAC cells expressing the full G-protein biosensor. (**B**) PDAC cells pre- and post-addition of starvation medium. (**C**) PDAC cells pre- and post-serum stimulation. Signal intensity colour bar from 0 to 450 arbitrary intensity units. Lifetime colour bar from 2000 to 3500 picoseconds. Scale bar, 50 μm. (Note that for quantification, only segmented cell membranes were used).

Overall, we draw three conclusions from these data. Firstly, FLIM-FRET yields quantitative data where only the donor fluorophore is excited (in this case, mNeonGreen), since the output is a lifetime map (Figure 3), where behind each pixel lies a lifetime value extracted after data fitting. Transparency in data fitting parameters is crucial whenever a FLIM approach is chosen, so the details described in the Methods section are particularly important. Secondly, this FLIM-based technique is sensitive enough to detect the small conformational changes that take place upon Gαi- protein activation. As mentioned in the Introduction, Gαi activation does not require large-scale structural changes like a complete subunit dissociation, which may occur in most other Gα subunits, such as Gαq. Instead, upon activation, Gαi and the corresponding Gβγ undergo a more subtle structural rearrangement [16,17,18]. Thirdly, the mNeonGreen-mCherry G-protein biosensor is functional on the plasma membrane of live PDAC cells and responds to external stimuli (LPA or FCS), as expected. Finally, our experiments using the LPAR1/3 inhibitor Ki16425 reproduce previous findings reporting on LPA signalling through LPAR1/3 and the Gi heterotrimer [20,23,45,46].

**Figure 4:**
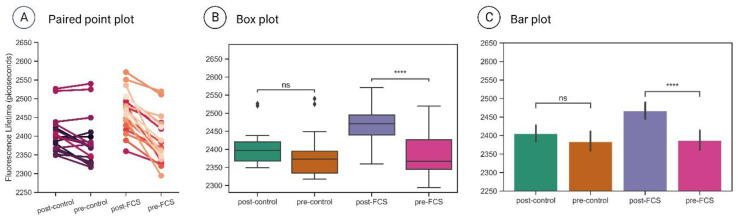
Different approaches to FLIM data visualisation. Data from this work were used here for demonstration purposes. (**A**) Paired point plot example. (**B**) Box plot example. (**C**) Bar plot example. The plots were created using Python and the pandas, matplotlib, statannot and seaborn packages. Note that statistical annotations are shown here for demonstration purposes only. (****: 1.00 × 10^−4^ < *p* ≤ 1.00 × 10^−5^); ns = not significant.

An important aspect of FLIM-FRET datasets is the manner in which they are visualised and presented to the reader. In Figure 4, the three most common ways FLIM-FRET data are reported in the literature are illustrated using one experimental sample from this study as an example. The paired point plot (Figure 4A) makes it immediately obvious to the reader which cells responded to the stimulation. The steepness of the line connecting a datapoint/cell pre- and post-stimulation also allows for a visual estimation of the magnitude of change in fluorescence lifetime. One disadvantage of this visualisation method is that the overlap in datapoints partially obscures their distribution. The box (Figure 4B) and bar plots (Figure 4C), however, suffer from inherent limitations that amount to a major disadvantage. In both cases, the visual cue of lifetime change per cell is lost, which means that the changes can only be seen in the whole population. As is clear in Figure 4B,C, this makes the change far less comprehensible to the reader. The numbers and distributions of datapoints are also lost (even if the former is given in the figure legend). The bar plot is by far the most problematic option, since it lacks the box plot’s whiskers and box dimensions that give a sense, albeit limited, of data distribution.

Instead, we suggest exploiting the freely available SuperPlotsofData web tool developed by Goedhart (2021) [26], alongside the paired point plots, which would give information on the absolute lifetime numbers and a visual cue on the steepness of change. SuperPlotsofData effectively remedies the drawbacks of the other limited presentation methods discussed here that may risk misleading interpretations. This tool, which may be directly accessed through a web browser, presents additional advantages since it is easy to use, and it does not require any coding experience, which was necessary for the generation of the plots in Figure 4 (Python packages such as pandas, matplotlib and seaborn). It may also be downloaded as an R package and run on the user’s own computer. Lifetime maps can also be useful; however, a colour change between the pre- and post-stimulation frames indicative of lifetime change is not always detectable by eye.

### 3.3. LPA as a Dominant Component of Serum

Earlier work on chemotaxis to serum [19] indicated that—for melanoma cells, at least—LPA is the predominant stimulus that steers cell migration.

**Figure 5:**
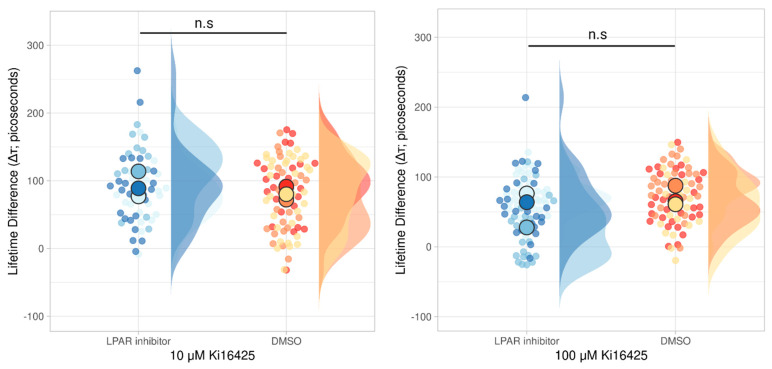
The LPA receptor inhibitor (Ki16425) cannot prevent G-protein activation after serum stimulation in PDAC cells even at high concentrations. Graph showing a lifetime increase after serum stimulation in cell samples treated with Ki16425 or vehicle (DMSO). Samples were treated with either 10 μΜ (**left** panel) or 100 μΜ (**right** panel) Ki16425. The independent *t*-test was used to calculate statistical significance with the statannot Python package (n.s: not significant). The data visualisation web tool SuperPlotsofData [26] was used to present the data. The different colours used show separate replicates, with the larger dot indicating the mean. Distribution per replicate is also shown. LPA; lysophosphatidic acid. DMSO; dimethylsulfoxide.

The inhibitor experiments presented earlier also suggested that LPAR1/3 were the key receptors. This was a surprise, because serum contains so many potential chemoattractants, including growth factors like PDGF and EGF and other lipid signals. Since we validated the system combining an mNeonGreen-mCherry G-protein biosensor with FLIM-FRET, we investigated whether LPA was a dominant component in serum for PDAC cells. Similarly, we were interested in whether Gαi3 activation can be triggered by serum signalling moieties other than LPA. To that end, we incubated biosensor-transfected PDAC cells with either the LPAR1/3 inhibitor Ki16425 or vehicle (DMSO) and stimulated both with serum. The results in Figure 5 show that the biosensor was activated in both conditions (approx. an average 100 picosecond lifetime increase), even when 100 μM Ki16425 was used. This was somewhat unexpected since Muinonen-Martin et al. (2014) [19] showed a complete arrest of chemotactic migration after treating metastatic melanoma cells with the same inhibitor. 

## 4. Discussion

The identification of optimal fluorescent protein pairs has been an ongoing challenge within the field of FRET applications [2,47,48]. Since the development of mTurquoise2 [33], the mTurquoise2-mVenus pair has been widely used for live cell FRET applications. However, in our hands, this FP pair has not met the brightness standards that have been reported previously [32,34]. In the field of fluorescent protein biochemistry, the molecular brightness of a fluorophore is typically described by multiplying its extinction coefficient with its quantum yield and often divided by 1000 [49]. Even at the time when mTurquoise2 was first described, mTFP1 and mCerulean3 were known to be brighter [50,51]. The measured brightness (see FPBase, https://www.fpbase.org; accessed 22 February 2024) for mTurquoise2, mCerulean3 and mTFP1 is 27.9, 34.8 and 54.4, respectively. This compares with a brightness of 92.8 for green fluorescent proteins such as mNeonGreen (FPBase; [52]). Using a 2-photon TCSPC FLIM system, we showed that mNeonGreen-mCherry has an approximately four-fold brighter signal than the mTurquoise2-mVenus pair. 

Moreover, mTurquoise2 is more blue-shifted than mNeonGreen, and its optimal two- photon excitation occurs at a shorter wavelength (890 nm vs. 970 nm for mNeonGreen), meaning that the laser needed for the excitation of the former is more energetic, leading to a greater rate of photodamage and, thus, more rapid cell death. One final complication with the mTurquoise2-mVenus pair is the concern of intermolecular dimers forming. Both proteins are derived from *Aequorea victoria* and may weakly tend towards dimerization. Although this can in some cases be regarded as beneficial for FRET probes, when it adds stability that maximises FRET efficiency, it can also cause artifacts when measuring FRET changes [53,54]. Fluorescent protein heterodimerisation could be a source of noise, confounding the resulting data, as an additional dimeric state exhibiting FRET would be present. This could pose problems for Gαi-based biosensors since, when activated, Gαi subunits are known to not completely dissociate from the Gβγ dimer but have a more subtle structural change as a complex [16,17,18].

During the G-protein biosensor optimisation process, we also found data visualization to be a crucial aspect of interpreting complex FLIM-FRET data. The lifetime difference post-stimulation of a sample is not always discernible by eye when viewing lifetime maps; thus, the proper quantification of data comparing each type of sample is necessary. We strongly recommend SuperPlotsofData [26] accompanied by paired point plots for two main reasons. First, this manner of data visualisation expresses confidence in the soundness of the data. Second, the experimental transparency is highlighted since the natural biological variability that characterises cell signalling is not obscured. Along with the reporting of an effect size measure (such as Cohen’s d), these recommended best practices that promote experimental data transparency are not necessarily restricted to FLIM-FRET data and may also apply to other research approaches.

Finally, the optimised and validated mNeonGreen-mCherry G-protein biosensor allowed us to interrogate whether LPA is the main component of FCS that activates the Gαi-coupled LPAR1/3. This hypothesis was based on previous findings by [19], who found that there was a decrease in the speed and directionality of melanoma cells migrating in serum when LPA signalling was blocked with the Ki16425 inhibitor. Perhaps surprisingly, we found that this LPAR1/3 inhibitor could not suppress the activation of the biosensor in response to stimulation from serum components. Two main factors could explain this discrepancy. First, it could be due to differences between the melanoma and PDAC cell lines used; the melanomas could express a narrower range of receptors than the PDAC cells. Alternatively, the difference could be due to the different experiments (pre- and post-simulation measurements of G-protein activation by FLIM-FRET vs. a migration assay). Muinonen-Martin et al. (2014) [19] and Juin et al. (2019) [20] showed the effect of LPA on migration by comparing the migration of melanoma and PDAC cells, respectively, in the presence or absence of the Ki16425 inhibitor. Additionally, Juin et al. (2019) [20] reported on the rapid rate of LPAR internalisation and trafficking upon LPA stimulation, suggesting the continuation of downstream LPAR signalling to RhoA. The two studies show that, upon addition of the inhibitor, melanoma cells often halt migration almost entirely and lose their directionality, unlike the PDAC cells that continue moving while lacking direction. Taken together, this suggests that while melanoma cells are dependent on LPAR1/3-mediated signalling for movement induction, the PDAC cells’ dependence lies only in the directionality LPA provides as a chemotactic signal. In other words, LPA may control the directionality of both cell types but only affects the migration speed of melanoma cells. While also considering the possibility of the two different cell lines having a different complement of receptors or different G-protein isoforms coupling to LPAR1/3, we could infer that in PDAC cells, there are additional pathways activating Gαi3 other than LPAR1/3. This appears likely given the particularly complex composition of serum [55,56]. Therefore, PDAC cells may express more Gαi3-coupled receptors that respond to non-LPA serum components, thus activating our G-protein biosensor. We believe two possible candidates could be sphingosine-1-phosphate (S1P) or platelet-activating factor (PAF) and their cognate GPCRs [57,58]. Finally, although unlikely given the sum of evidence provided here, the LPAR1/3-Gαi3 signalling axis could play more of a contributing rather than a starring role in the context of PDAC chemotaxis.

### Limitations of the Study

TCSPC FLIM is generally thought of as a slow imaging technique, with acquisition times that may reach several minutes per frame. Despite that, our instrumentation allows for relatively short acquisition times of approximately 14.4 s. Commercially available systems, such as the Leica TCS-SP8 FALCON confocal microscope [8], can be several-times faster. Our system, however, is limited with respect to temporal resolution since it lacks an automated z-plane focus system. Thus, in practical terms, after every sample stimulation, and before acquiring a post-stimulation image, each field of view must be manually refocused. Nevertheless, this is not particularly limiting unless timelapse imaging of moving cells is desired.

It is important to note that there are many ways of fitting FLIM data and extracting lifetimes [3]. However, by using FLIMfit, the user is restricted to only two options for the fitting algorithm (Maximum Likelihood or Variable Projection). All fitting methods are associated with certain advantages and disadvantages, with Maximum Likelihood Estimation being the preferred method, specifically for TCSPC samples, especially if the samples are not particularly bright [59]. This limitation in available fitting algorithms, in practice, only has the potential of becoming problematic when tissue samples are imaged. In this case, a stretched exponential fit would be best [28]. The breadth of options available during lifetime extraction in FLIMfit might also pose a challenge for inexperienced users. Certain options have rare use cases and are open to abuse. For example, the “Estimate IRF Shift” function should only be used under exceptional circumstances where an Instrument Response Function (IRF) could not be acquired (as discussed in the FLIMfit Documentation, Release 4.11.1). Used inappropriately, this function understates the divergence of the real intensity decay from the model fit.

In our experience, the primary challenge was associated with limitations that concern the need for cells to be transiently transfected with the G-protein biosensor. Despite this, we were determined to use primary mouse PDAC cells [25], instead of HEK293 or MDCK cells, so that this system could be used to answer complex biological questions. Unfortunately, creating stable cell lines to conduct longer-term experiments, for example, 48 h long migration or invasion assays, is not an option since the level of expression of the biosensor would not be high enough compared to that of transient transfections. Thus, acceptable levels of expression are necessary, even if the donor fluorophore is bright or the laser power capabilities of the microscope are high, without compromising cell behaviour and viability. Realistically, it is hard to predict which cell lines of interest would tolerate the overexpression of the G-protein complex. For example, we did attempt transiently transfecting the melanoma cell lines used in the study by Muinonen-Martin et al. (2014) [19], but we were unable to obtain sufficient levels of signal that would allow for accurate lifetime measurements. Worse still, this overexpression may have adverse effects on the normal function of the probe, meaning the results obtained do not reflect physiological behaviour. Nonetheless, these considerations apply to all experiments that depend on protein overexpression.

## Data Availability

Primary data are available on request from the corresponding author.

## References

[1] Förster T. (2012). Energy migration and fluorescence. J. Biomed. Opt..

[2] Bajar B.T., Wang E.S., Zhang S., Lin M.Z., Chu J. (2016). A Guide to Fluorescent Protein FRET Pairs. Sensors.

[3] Datta R., Heaster T.M., Sharick J.T., Gillette A.A., Skala M.C. (2020). Fluorescence lifetime imaging microscopy: Fundamentals and advances in instrumentation, analysis, and applications. J. Biomed. Opt..

[4] Peter M., Ameer-Beg S.M. (2004). Imaging molecular interactions by multiphoton FLIM. Biol. Cell.

[5] Van Munster E.B., Gadella T.W.J. (2005). Fluorescence Lifetime Imaging Microscopy (FLIM). Adv. Biochem. Eng. Biotechnol..

[6] Wallrabe H., Periasamy A. (2004). Imaging protein molecules using FRET and FLIM microscopy. Curr. Opin. Biotechnol..

[7] Adjobo-Hermans M.J., Goedhart J., van Weeren L., Nijmeijer S., Manders E.M., Offermanns S., Gadella T.W. (2011). Real-time visualization of heterotrimeric G protein Gq activation in living cells. BMC Biol..

[8] Harkes R., Kukk O., Mukherjee S., Klarenbeek J., Broek B.v.D., Jalink K. (2021). Dynamic FRET-FLIM based screening of signal transduction pathways. Sci. Rep..

[9] Nobis M., McGhee E.J., Morton J.P., Schwarz J.P., Karim S.A., Quinn J., Edward M., Campbell A.D., McGarry L.C., Evans T.J. (2013). Intravital FLIM-FRET Imaging Reveals Dasatinib-Induced Spatial Control of Src in Pancreatic Cancer. Cancer Res..

[10] McCullock T.W., MacLean D.M., Kammermeier P.J. (2020). Comparing the performance of mScarlet-I, mRuby3, and mCherry as FRET acceptors for mNeonGreen. PLoS ONE.

[11] Shaner N.C., Lambert G.G., Chammas A., Ni Y., Cranfill P.J., Baird M.A., Sell B.R., Allen J.R., Day R.N., Israelsson M. (2013). A bright monomeric green fluorescent protein derived from Branchiostoma lanceolatum. Nat. Methods.

[12] Warren S.C., Margineanu A., Alibhai D., Kelly D.J., Talbot C., Alexandrov Y., Munro I., Katan M., Dunsby C., French P.M.W. (2013). Rapid Global Fitting of Large Fluorescence Lifetime Imaging Microscopy Datasets. PLoS ONE.

[13] Chavez-Abiega S., Grönloh M.L.B., Gadella T.W.J., Bruggeman F.J., Goedhart J. (2022). Single-cell imaging of ERK and Akt activation dynamics and heterogeneity induced by G-protein-coupled receptors. J. Cell Sci..

[14] Chavez-Abiega S., Goedhart J., Bruggeman F.J. (2019). Physical biology of GPCR signalling dynamics inferred from fluorescence spectroscopy and imaging. Curr. Opin. Struct. Biol..

[15] Van Unen J., Stumpf A.D., Schmid B., Reinhard N.R., Hordijk P.L., Hoffmann C., Gadella Jr T.W., Goedhart J. (2016). A new generation of FRET sensors for robust measurement of Gαi1, Gαi2 and Gαi3 activation kinetics in single cells. PLoS ONE.

[16] Bondar A., Lazar J. (2014). Dissociated GαGTP and Gβγ protein subunits are the major activated form of heterotrimeric Gi/o proteins. J. Biol. Chem..

[17] Bünemann M., Frank M., Lohse M.J. (2003). Gi protein activation in intact cells involves subunit rearrangement rather than dissociation. Proc. Natl. Acad. Sci. USA.

[18] Frank M., Thümer L., Lohse M.J., Bünemann M. (2005). G Protein Activation without Subunit Dissociation Depends on a Gαi-specific Region. J. Biol. Chem..

[19] Muinonen-Martin A.J., Susanto O., Zhang Q., Smethurst E., Faller W.J., Veltman D.M., Kalna G., Lindsay C., Bennett D.C., Sansom O.J. (2014). Melanoma Cells Break Down LPA to Establish Local Gradients That Drive Chemotactic Dispersal. PLoS Biol..

[20] Juin A., Spence H.J., Martin K.J., McGhee E., Neilson M., Cutiongco M.F., Gadegaard N., Mackay G., Fort L., Lilla S. (2019). N-WASP Control of LPAR1 Trafficking Establishes Response to Self-Generated LPA Gradients to Promote Pancreatic Cancer Cell Metastasis. Dev. Cell.

[21] Chuong M.D., Herrera R., Ucar A., Aparo S., De Zarraga F., Asbun H., Jimenez R., Asbun D., Narayanan G., Joseph S. (2023). Causes of Death Among Patients with Initially Inoperable Pancreas Cancer After Induction Chemotherapy and Ablative 5-fraction Stereotactic Magnetic Resonance Image Guided Adaptive Radiation Therapy. Adv. Radiat. Oncol..

[22] Gohla A., Harhammer R., Schultz G. (1998). The G-protein G13 but Not G12 Mediates Signaling from Lysophosphatidic Acid Receptor via Epidermal Growth Factor Receptor to Rho. J. Biol. Chem..

[23] Stähle M., Veit C., Bachfischer U., Schierling K., Skripczynski B., Hall A., Gierschik P., Giehl K. (2003). Mechanisms in LPA-induced tumor cell migration: Critical role of phosphorylated ERK. J. Cell Sci..

[24] Auciello F.R., Bulusu V., Oon C., Tait-Mulder J., Berry M., Bhattacharyya S., Tumanov S., Allen-Petersen B.L., Link J., Kendsersky N.D. (2019). A Stromal Lysolipid–Autotaxin Signaling Axis Promotes Pancreatic Tumor Progression. Cancer Discov..

[25] Morton J.P., Timpson P., Karim S.A., Ridgway R.A., Athineos D., Doyle B., Jamieson N.B., Oien K.A., Lowy A.M., Brunton V.G. (2009). Mutant p53 drives metastasis and overcomes growth arrest/senescence in pancreatic cancer. Proc. Natl. Acad. Sci. USA.

[26] Goedhart J. (2021). SuperPlotsOfData—A web app for the transparent display and quantitative comparison of continuous data from different conditions. Mol. Biol. Cell.

[27] Talbot C.B., Patalay R., Munro I., Warren S., Ratto F., Matteini P., Pini R., Breunig H.G., König K., Chu A.C. (2011). Application of ultrafast gold luminescence to measuring the instrument response function for multispectral multiphoton fluorescence lifetime imaging. Opt. Express.

[28] Siegel J., Lee K.B., Webb S.E., Leveque-Fort S., Cole M.J., Jones R., Dowling K., French P.M., Lever M.J. (2001). Application of the stretched exponential function to fluorescence lifetime imaging of biological tissue. Biophys. J..

[29] Brownlee J. (2019). Effect Size Measures in Python. https://machinelearningmastery.com/effect-size-measures-in-python/.

[30] Cohen J. (1988). Statistical Power Analysis for the Behavioral Sciences.

[31] Lakens D. (2013). Calculating and reporting effect sizes to facilitate cumulative science: A practical primer for t-tests and ANOVAs. Front. Psychol..

[32] Klarenbeek J., Goedhart J., van Batenburg A., Groenewald D., Jalink K. (2015). Fourth-Generation Epac-Based FRET Sensors for cAMP Feature Exceptional Brightness, Photostability and Dynamic Range: Characterization of Dedicated Sensors for FLIM, for Ratiometry and with High Affinity. PLoS ONE.

[33] Goedhart J., von Stetten D., Noirclerc-Savoye M., Lelimousin M., Joosen L., Hink M.A., Van Weeren L., Gadella T.W.J., Royant A. (2012). Structure-guided evolution of cyan fluorescent proteins towards a quantum yield of 93%. Nat. Commun..

[34] Mastop M., Bindels D.S., Shaner N.C., Postma M., Gadella T.W.J., Goedhart J. (2017). Characterization of a spectrally diverse set of fluorescent proteins as FRET acceptors for mTurquoise2. Sci. Rep..

[35] Shaner N.C., Campbell R.E., Steinbach P.A., Giepmans B.N., Palmer A.E., Tsien R.Y. (2004). Improved monomeric red, orange and yellow fluorescent proteins derived from *Discosoma* sp. red fluorescent protein. Nat. Biotechnol..

[36] Shaner N.C., Steinbach P.A., Tsien R.Y. (2005). A guide to choosing fluorescent proteins. Nat. Methods.

[37] Coucke Q., Parveen N., Fernández G.S., Qian C., Hofkens J., Debyser Z., Hendrix J. (2023). Particle-based phasor-FLIM-FRET resolves protein-protein interactions inside single viral particles. Biophys. Rep..

[38] Scott B.L., Hoppe A.D. (2015). Optimizing fluorescent protein trios for 3-Way FRET imaging of protein interactions in living cells. Sci. Rep..

[39] Balleza E., Kim J.M., Cluzel P. (2018). Systematic characterization of maturation time of fluorescent proteins in living cells. Nat. Methods.

[40] Ridley A.J., Paterson H.F., Johnston C.L., Diekmann D., Hall A. (1992). The Small GTP-Binding Protein rac Regulates Growth Factor-Induced Membrane Ruffling. Cell.

[41] Jongsma M., Matas-Rico E., Rzadkowski A., Jalink K., Moolenaar W.H. (2011). LPA is a chemorepellent for B16 melanoma cells: Action through the cAMP-elevating LPA 5 receptor. PLoS ONE.

[42] Pagès C., Simon M.-F., Valet P., Saulnier-Blache J.S. (2001). Lysophosphatidic acid synthesis and release. Prostaglandins Other Lipid Mediat..

[43] Plonsky L., Oswald F.L. (2014). How Big Is “Big”? Interpreting Effect Sizes in L2 Research. Lang. Learn..

[44] Sullivan G.M., Feinn R. (2012). Using Effect Size—Or Why the P Value Is Not Enough. J. Grad. Med. Educ..

[45] Fukushima N., Kimura Y., Chun J. (1998). A single receptor encoded by vzg-1lp A1 edg-2 couples to G proteins and mediates multiple cellular responses to lysophosphatidic acid. Proc. Natl. Acad. Sci. USA.

[46] Ohta H., Sato K., Murata N., Damirin A., Malchinkhuu E., Kon J., Kimura T., Tobo M., Yamazaki Y., Watanabe T. (2003). Ki16425, a Subtype-Selective Antagonist for EDG-Family Lysophosphatidic Acid Receptors. Mol. Pharmacol..

[47] Martin K.J., McGhee E.J., Schwarz J.P., Drysdale M., Brachmann S.M., Stucke V., Sansom O.J., Anderson K.I. (2018). Accepting from the best donor; analysis of long-lifetime donor fluorescent protein pairings to optimise dynamic FLIM-based FRET experiments. PLoS ONE.

[48] Shcherbo D., Souslova E.A., Goedhart J., Chepurnykh T.V., Gaintzeva A., Shemiakina I.I., Gadella T.W., Lukyanov S., Chudakov D.M. (2009). Practical and reliable FRET/FLIM pair of fluorescent proteins. BMC Biotechnol..

[49] Gaigalas A., Li L., Henderson O., Vogt R., Barr J., Marti G., Weaver J., Schwartz A. (2001). The development of fluorescence intensity standards. J. Res. Natl. Inst. Stand. Technol..

[50] HAi H.W., Henderson J.N., Remington S.J., Campbell R.E. (2006). Directed evolution of a monomeric, bright and photostable version of Clavularia cyan fluorescent protein: Structural characterization and applications in fluorescence imaging. Biochem. J..

[51] Markwardt M.L., Kremers G.-J., Kraft C.A., Ray K., Cranfill P.J.C., Wilson K.A., Day R.N., Wachter R.M., Davidson M.W., Rizzo M.A. (2011). An Improved Cerulean Fluorescent Protein with Enhanced Brightness and Reduced Reversible Photoswitching. PLoS ONE.

[52] Lambert T.J. (2019). FPbase: A community-editable fluorescent protein database. Nat. Methods.

[53] Karpova T.S., Baumann C.T., He L., Wu X., Grammer A., Lipsky P., Hager G.L., McNally J.G. (2003). Fluorescence resonance energy transfer from cyan to yellow fluorescent protein detected by acceptor photobleaching using confocal microscopy and a single laser. J. Microsc..

[54] Miyawaki A., Tsien R.Y. (2000). Monitoring protein conformations and interactions by fluorescence resonance energy transfer between mutants of green fluorescent protein. Methods Enzymol..

[55] Else P.L. (2019). The highly unnatural fatty acid profile of cells in culture. Prog. Lipid Res..

[56] Chelladurai K.S., Rajagopalan K., Yesudhason B.V., Venkatachalam S., Mohan M., Vasantha N.C., Christyraj J.R.S.S. (2021). Alternative to FBS in animal cell culture—An overview and future perspective. Heliyon.

[57] Brown S.L., Jala V.R., Raghuwanshi S.K., Nasser M.W., Haribabu B., Richardson R.M. (2006). Activation and Regulation of Platelet-Activating Factor Receptor: Role of Gi and Gq in Receptor-Mediated Chemotactic, Cytotoxic, and Cross-Regulatory Signals. J. Immunol..

[58] Liu S., Paknejad N., Zhu L., Kihara Y., Ray M., Chun J., Liu W., Hite R.K., Huang X.-Y. (2022). Differential activation mechanisms of lipid GPCRs by lysophosphatidic acid and sphingosine 1-phosphate. Nat. Commun..

[59] Becker W. (2021). The Bh TCSPC Handbook.

